# Growth Monitoring and Promotion Index Development: A Novel Approach

**DOI:** 10.3390/healthcare11142011

**Published:** 2023-07-12

**Authors:** Shamiso Alice Moyo, Ntsieni Stella Mashau, Lufuno Makhado

**Affiliations:** 1 Department of Public Health, Faculty of Health Sciences, University of Venda, Thohoyandou 0950, South Africa; 2 Office of the Executive Dean, Faculty of Health Sciences, University of Venda, Thohoyandou 0950, South Africa

**Keywords:** growth monitoring and promotion index, barriers, facilitators, community health worker, children under five, Zimbabwe

## Abstract

Background: There are few growth monitoring and promotion indexes, and currently none of them include any metrics that measure caregiver behaviours. No index to date combines the metrics of both community health worker activeness and caregiver barriers and facilitators towards growth monitoring and promotion (GMP). This study developed a new growth monitoring and promotion index and validated it using the Delphi Technique. Methods: The study began with phase 1, which was a scoping review of the literature on GMP indexes. Phase 2 involved a community health worker (CHW) survey which explored the process of GMP within the Umguza health system, and determined how knowledge of GMP by CHWs translated to frequency of activities. A barrier analysis was also conducted with caregivers of children under five to determine the barriers and facilitators towards GMP attendance by caregivers. Phase 3 was the construction of the index along with its validation, using the Delphi Technique where fifteen experts within the health and nutrition sector were consulted to analyse the constructs/variables of the index. Results: A growth monitoring and promotion index was developed and validated by several technical experts in the health and nutrition sector in Zimbabwe. Conclusions: A new index has been developed to improve the quality of growth monitoring and promotion activities within the communities.

## 1. Background

Growth monitoring and promotion (GMP) is both a preventive and promotive activity that is used by health workers to monitor the growth of children. It helps in the prevention of child malnutrition through early detection of any growth faltering [1] and reduces the morbidity and mortality of children [2]. The first 2 years of a child’s life, also known as the first 1000 days, are extremely important. These years have been described as a critical “window of opportunity” meant to ensure appropriate child growth through optimal feeding [3]. GMP, if not practiced appropriately in the first 2 years, can make children more prone to malnutrition [4].

In Zimbabwe, GMP is an important part of the nutrition surveillance system which operates from the national level right down to the community level [5]. At the community level, GMP is supported by community health workers (CHWs). A CHW is “any health worker carrying out functions related to health care delivery; trained in some way in the context of the intervention, and having no formal professional or paraprofessional certificate or degree in tertiary education.” [6,7]. In this study, the CHW being referred to by the definition is equivalent to the village health worker (VHW) in Zimbabwe who serves as the conduit of GMP between the caregivers of children under five (CU5) and the health facilities. The CHWs monitor the nutrition and health status of CU5 through the collection of weight, mid-upper arm circumference (MUAC), age-appropriate nutrition counselling, and vitamin A supplementation, among many other activities. The data collected are submitted to the health facilities using the T-forms and entered into the District Health Information System (DHIS2) [5].

The challenges of GMP are two-pronged, those affecting the CHWs and those affecting the caregivers of CU5, and result in a vicious cycle. On the part of the CHWs, they are meant to be volunteers working for 2–3 half days a week. However, findings by the Zimbabwe Parliamentary Portfolio Committee on Health in 2017 found that the CHWs were now working throughout the week including at night. This then led to the realisation that they were working 40 h weeks [7]. This certainly has a bearing on the activity levels of CHWs with regard to full-time roles versus part-time roles.

Among some of the challenges faced by caregivers of CU5 is the aspect of distance to the GMP site [8]. Generally, caregivers do not want to walk long distances to seek health care services. This was noted as a barrier towards GMP attendance [9], and furthermore, such barriers create negative attitudes towards attendance to GMP by caregivers. A study conducted by Mphasha et al. [10] in Polokwane, South Africa, explored the attitudes of caregivers towards GMP. They concluded that the inconsistent availability of GMP services affected caregiver attendance to GMP. This then points back to the CHWs and the challenges they face, hence the vicious cycle which is affecting the overall quality of GMP.

From the protocol by Moyo et al. [11], a data challenge in the Umguza district, in Matabeleland North Province of Zimbabwe, was identified where CHWs were not compiling GMP statistics. This rendered the data poor-quality and of little use. Furthermore, what exists in the DHIS2 are indicators such as “the number of children weighed” which do not tell us about the quality of GMP activities. By quality, we refer to aspects of whether caregivers of CU5 attended GMP in the first place along with any barriers or facilitators they may face concerning GMP. Some studies have even shown that healthcare workers mainly emphasized weighing the children in the absence of counselling for mothers/caregivers on children’s feeding based on the growth curve [12], and other studies conducted on GMP practice reported that counselling offered to mothers/caregivers during GMP service is weak [13,14]. The protocol by Moyo et al. [11] also adapted a conceptual framework by Ashworth et al. [14]. This framework showed how these different elements (caregivers of CU5, CHWs) were interlinked and ultimately affect the health and nutritional status of CU5. These examples serve to illustrate how important it is to consider other GMP elements in determining the quality and overall classification of GMP activities within a community.

In the build-up to the GMP index (GMPI), literature was reviewed which highlighted a gap with regard to the types and number of child health indexes currently existing. No index combines the metrics of both CHW performance and caregiver barriers and facilitators towards GMP [9]. It is against this background that this research sought to construct a GMPI along with validating its construction.

The study took place in Umguza district, found in Matabeleland North Province, Zimbabwe. The district has a total population of 113,265 [15] and 25 primary health facilities. There is a mixed population of both rural and peri-urban populations who mainly rely on agriculture-based trades.

## 2. Methods

The study was conducted in three phases, where phase 1 was a scoping literature review; phase 2, a CHW survey and barrier analysis; and phase 3, the development of the index.

### 2.1. Phase 1

The objectives of phase 1 were to explore and analyse knowledge gaps about GMP activities and to describe key characteristics related to GMP index development. To investigate these, a scoping literature review was conducted using the Rodgers Concept Analysis Framework and identified antecedents, attributes, and consequences of GMP activities worldwide. The review included peer-reviewed and published quantitative and qualitative studies about GMP activities up to April 2022. Excluded were GMP activities conducted at the primary health facilities. The AMSTAR tool was used for quality checks. A total of 535 articles were selected; however, only 316 were found to be relevant to the study. Of these, 80 articles met the inclusion criteria, and only 25 were included in the final analysis.

### 2.2. Phase 2

The objectives of phase 2 were to explore the process of GMP within the Umguza health system as conducted by CHWs, to determine how knowledge of growth monitoring and promotion by CHWs translates to a frequency of activities, to determine the barriers and facilitators towards GMP attendance by caregivers of CU5, and to identify sustainable best practices among CHWs consistently conducting growth monitoring and promotion activities. To achieve these, this phase used a sequential mixed-methods design. A CHW survey was conducted initially, where a census of 186 CHWs in the Umguza district were interviewed. The survey, being quantitative in nature, used a questionnaire to collect data which were then analysed with SPSS version 25. Ethical considerations were justice, beneficence, confidentiality, and anonymity.

A barrier analysis (BA) then followed with the target population of the caregivers of CU5. The BA was both explorative and descriptive, and it was based on the Health Belief Model and Theory of Reasoned Action. Included were 45 caregivers of CU5 who attend GMP monthly (doers) and 45 caregivers of CU5 who do not attend monthly GMP (non-doers), to make a total of 90 caregivers. The standard BA questionnaire was used to collect the data, while the standard BA tabulation sheet was used for data analysis. From the results obtained, focus group discussions were held with caregivers of CU5 who do not attend monthly GMP activities. Data collected were transcribed verbatim and analysed using ATLAS.ti 8.

### 2.3. Phase 3

The objectives of phase 3 were to construct a GMP index along with guidelines for its usage and to test and validate the GMP index. The GMP index development process was thus conducted in three steps: (1) variable selection, (2) examining empirical relationships of the variables and thereafter combining them into an index, and (3) index validation.

#### 2.3.1. Step 1: Variable Selection

From the scoping literature review results, the antecedent CHW activeness, the attribute knowledge, attitude and practices of caregivers, and the consequence of improved child health outcomes were identified as important with regard to GMP activities. Literature also showed how they all had a direct effect on GMP activities. Having investigated these aspects during phase 2, it was thus necessary to relate them to the index in the form of an equation. The starting point was defining the index and how it was a function of its variables.

In this study, the GMP index (GMPI) was defined as the quality of GMP activities as conducted by CHWs. It is a function of CHW activeness, the magnitude of caregiver barriers to GMP, and the magnitude of caregiver facilitators towards GMP. The barriers are impediments and weigh down the index. In linking the variables into one, an equation was developed by the principal investigator. It illustrated GMPI as a sum of its variables because the variables of CHW activeness, magnitude of barriers to GMP, and magnitude of facilitators to GMP directly affect the GMPI. The equation was thus set as follows:

GMPI = X + Y − Z(1)

where

X = CHW activeness
Y = caregiver facilitators of GMP
Z = caregiver barriers to GMP.

The variables X, Y, and Z were determined as follows:

- X (CHW activeness)

CHW activeness was defined as the number of hours per week spent on the various CHW activities by the CHWs (CHW activities in their broad sense and not entirely just aligned to GMP activities).

- Y (caregiver facilitators of GMP)

Facilitators were defined as any entity that can help a person adopt and practice a specific behaviour. The variable Y was thus defined as the magnitude of facilitators identified. It has a positive effect on the GMPI as it improves the quality of GMP activities.

- Z (caregiver barriers to GMP)

Barriers were defined as any hindrances that prevent a person from adopting and practicing a specific behaviour. The variable Z was thus defined as the magnitude of barriers identified. It has a negative effect on the GMPI as the barriers are obstructions that hinder the implementation of quality GMP activities.

#### 2.3.2. Step 2: Examination of Empirical Relationships among Variables and Combining Them into an Index

During this step, a review of the data analysis from the CHW survey and barrier analysis of the caregivers of CU5 was conducted.

- CHW Activeness (X)

The objectives of the CHW survey were to explore the process of GMP in Umguza district as conducted by CHWs and to determine how knowledge of GMP translated to the frequency of their activities. The survey found that there was a significant relationship between the level of knowledge of GMP aspects and the frequency of GMP activities. The CHW survey also assessed the number of hours per week that the CHWs work, and this was selected as a measure of how active they were, hence variable X, i.e., CHW activeness. The number of hours per week ranged from a low of 2 h to a high of 40 h and a mean of 16.99 h. The question to the CHW read: How many hours per week are spent on CHW activities? A five-point Likert scale was then created to classify the different levels/types of CHW activeness as follows: least active (0–8 h); somewhat active (9–16 h); active (17–24 h); very active (25–32 h); and most active (33–40 h) [16].

- Caregiver facilitators (Y) and barriers (Z) to GMP

The barrier analysis (BA) was conducted to identify the barriers and facilitators towards GMP affecting caregivers of CU5 [17]. The methodology utilised the BA questionnaire which was administered to caregivers of CU5 (45 doers and 45 non-doers), and analysis was then conducted using the BA sheet. Those factors that were identified with *p* values of < 0.05 were included as the key barriers and facilitators [17]. From a meta-analysis by Carpenter [18], and a study by Orji et al. [19], which served as an extension to the Health Belief Model, it was seen that barriers emerged as the strongest predictors of behaviour, influencing the likelihood of an individual performing the targeted behaviour. It can thus be seen how much they affect the adoption of behaviours. From the study, four barriers were identified as being the distance to health facilities, time taken during GMP activities, transport costs, and family support, whilst distance to health facilities was the only identified facilitator and made up variables Z and Y.

Against this inductive reasoning, the most suitable approach was deemed as being the use of the Likert scales so as the be able to merge the variables X, Y, and Z into a GMPI. The method used was kept simple to be easily replicated at the program implementation or district level where the DHIS2 database is managed.

Likert scales were used for all three variables X, Y, and Z and were developed to classify each variable and subsequently quantify the GMPI itself.

#### 2.3.3. Step 3: Validation of the Index Using the Delphi Technique

The Delphi Technique was used in validating the GMPI. It is a cross-examination process whose aim is to obtain expert-based judgements about a topic of interest from experts [20,21,22]. The different experts were purposively drawn from varying backgrounds that covered academia, the Ministry of Health and Child Care (national, provincial, and district levels), policy development, nutrition program management specialists, and SBC specialists. They were selected on the basis that they would be able to provide rich insights and meaningfully critique the newly developed GMPI. They were given in-depth information about the GMPI and tasked with critiquing the variables identified that constitute the GMPI. They were given a four-week timeline to respond to their given task. Thereafter, meetings were held with each expert to discuss their expert-based judgements. The experts were able to draw on their experience within the health sector and/or knowledge from other types of studies and, in so doing, captured their consensus and enhanced the overall quality of the GMPI [21].

A total of fifteen experts participated, as shown in Table 1 below.

The opinions of the 15 experts were captured and are collectively outlined in the results section below.

### 2.4. Guidelines for GMPI Usage

The guidelines for the use of the GMPI were developed to guide GMP implementers on how to use the GMPI in their wards or districts. They outlined the variables in the GMPI, i.e., the source of the data and how they are measured. They are shown in the results section below.

## 3. Results

The GMPI itself is a culmination of phases 1 and 2 as outlined by the summary of the findings from phases 1 and 2 (Table 2) and the final development in phase 3. The first column in Table 2 shows a summary of findings from the scoping literature review which was phase 1. Columns 2 and 3 highlight the findings from the CHW survey and barrier analysis with caregivers of CU5 which was phase 2. The fourth column outlines a summary from merging phases 1 and 2. The variables, though seemingly different, are interconnected through the caregivers of CU5, CHWs, and GMP for CU5. 

### 3.1. Variable X: CHW Activeness

Using a five-point Likert scale, scores were assigned for each level of CHW activeness with the least active being assigned the lowest score of 1 and the most active being assigned a score of 5. This meant that the maximum score attained could only be 5 points for CHW activeness. Table 3 shows that almost half of the CHWs were moderately active, with only 35.5% of the CHWs being classified as active.

To develop a single score for all the CHWs, a weighted average score was used, i.e., (frequency × scores)/n.

The calculation thus follows: [(12 × 1) + (89 × 2) + (66 × 3) + (14 × 4) + (5 × 5)]/186 = 2.52 (2 decimal places).

The level of CHW activeness for this study was therefore 2.5.

### 3.2. Variable Y: Caregiver Facilitators of GMP

A three-point Likert scale was developed to classify the magnitude of facilitators of GMP (Table 2) identified from the barrier analysis. The ideal situation is that the presence of many facilitators creates an enabling environment for the caregivers of CU5 to attend GMP. The more facilitators, the higher the score on the Likert scale. A maximum score of 3 points was allocated for four or more facilitators based on the logic that the existence of many facilitators acts as a push towards GMP attendance, while the lowest score of 1 point was allocated for one or fewer facilitators. This is shown in Table 4 below.

This study identified one facilitator; hence, the overall score for variable Y is 1.

### 3.3. Variable Z: Caregiver Barriers to GMP

A three-point Likert scale was developed to classify the magnitude of the barriers identified in this study (Table 2) towards GMP from the barrier analysis. Based on the definition of barriers, it means that there were many hindrances to GMP in the Umguza district, and this is not the desirable situation. Hence, the more barriers, the less the caregivers will attend GMP activities. This variable weighs down on the GMP activities and is represented as negative on the equation. A score of 3 points was allocated for four or more barriers, while a score of 1 point was allocated for one or fewer barriers, as shown in Table 5 below.

This study identified four barriers; hence, the overall score for variable Z is 3.

### 3.4. GMPI for the Umguza District

From the equation developed from this study, GMPI = X + Y − Z, the maximum value of the GMPI is attained when the following conditions are met:

- CHW activeness (X) is classified as most active (Table 3) and score is 5;
- Caregiver facilitators of GMP (Y) are many (Table 4) and score is 3;
- Caregiver barriers to GMP (Z) are few (Table 5) and score is 1.

The maximum GMPI score is 7 from the formula calculation [5 + 3 − 1].

The minimum value of the GMPI is attained when the following conditions are met:

- CHW Activeness (X) is classified as least active (Table 3);
- Caregiver facilitators of GMP (Y) are few (Table 4);
- Caregiver barriers to GMP (Z) are many (Table 5).

For the classification of the GMPI, a three-point Likert scale was developed where each threshold was weighted equally to one decimal place [16], i.e., low (0–2.3), medium (2.4–4.7), and high (4.8–7) so as to be able to fully classify the GMPI as shown in Table 6.

From the results of this study, the GMPI is based on the equation GMPI = X + Y − Z where the variables have been outlined above as follows:

X = 2.5 CHW activeness
Y = 1 caregiver facilitators of GMP
Z = 3 caregiver barriers to GMP

The GMPI = 0.5 [2.5 + 1 − 3].

According to this study, GMPI is a measure of the quality of GMP activities, and thus it follows that the highest quality of GMP activities exists within a district or community when there are the following:

- Few barriers (Z);
- Many facilitators (Y);
- Most active CHWs (X).

Based on the GMPI score above, the quality of GMP activities in the Umguza district was found to be low.

### 3.5. Validation of the GMPI

**Figure 1:**
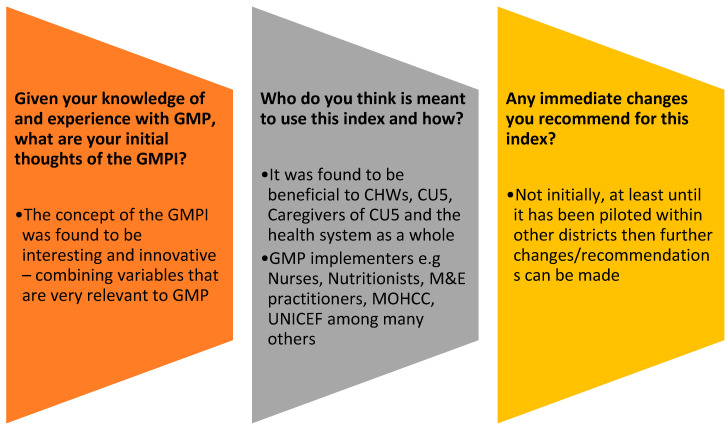
Opinions of technical experts from validation exercise.

An analysis of opinions from the technical experts is shown in Figure 1 below.

Based on the GMPI validation exercise, it was agreed that no immediate changes were to be made to the new GMPI. Recommendations were that the GMPI be tried out by the MOHCC and other implementers of GMP programs to ascertain its usability.

### 3.6. Guidelines on Usage of the GMPI

- The GMPI can be used by all implementers of GMP at either the district or ward level.
- It is best to use the GMPI initially when the routine GMP data indicate a low number of CU5 being assessed during GMP by CHWs. Such data can be obtained from the DHIS2.
- To use the GMPI, the different variables that constitute it must be measured. Their measurement is outlined below.

- CHW activeness (X).

This is measured through a survey of all CHWs in the ward or district. A questionnaire should be designed to include the number of hours volunteered by the CHWs per week, among other desired aspects. Once data are obtained, analysis can be conducted using SPSS or STATA (refer to Table 7 below).

In order to develop a single score for all the CHWs, a weighted average score should be used: (frequency × scores)/n.

II. Caregiver facilitators of GMP (Y)

A standard barrier analysis as developed by Kittle [17] is conducted to identify the number of facilitators affecting caregivers of CU5 within the ward or district. Once the number is identified, it is classified according to Table 8 below.

III. Caregiver barriers to GMP (Z)

A standard barrier analysis as developed by Kittle [17] is conducted to identify the number of barriers affecting caregivers of CU5 within the ward or district. Once the number is identified, it is classified according to Table 9 below.

D. Classification using the GMPI

From this study, GMPI is the sum of X + Y − Z. Table 10 below shows the GMPI thresholds and subsequent classification.

Hence, based on the total sum of the three variables, the GMPI will be found and classified as to whether it is low, medium, or high.

High implies that GMP activities as shown by the variables are performing well.

Medium implies that one or more of the variables are performing poorly and specific strategies to improve them need to be implemented.

Low implies that two or more of the variables are performing poorly and specific strategies to improve them need to be implemented.

## 4. Discussion

Having defined the GMPI as a measure of the quality of GMP activities has enabled GMP implementers to move away from the usual classifications which were based on the currently existing child health indexes such as weight-for-height. The scoping literature review by Moyo et al. [23] identified a few indexes that have been used for the GMP of children. The most common indexes are the WHO Growth Standards that only assess nutritional status. From this review, it was concluded that there was currently no index linking the behaviours of caregivers of CU5 towards GMP and CHW activities. Furthermore, the GMPI is designed to be used at the lowest level, i.e., village. The measurement of the GMPI at the village level will provide many timeous benefits for both CU5 and their caregivers. A study conducted in Nepal highlighted that when growth monitoring was brought to communities, there was an increased demand for clean water, home nutrition gardens, and income-generating projects [24]. This is against a long-standing background of caregivers failing to attend GMP sessions at health facilities due to high transport costs or other competing family-related responsibilities [14].

This brings to light the importance of CHWs, who are central to GMP activities at the community level. They collect the data that filter into the DHIS2. In the DHIS2, one of the indicators reflective of GMP is the number of children weighed per month. Caregivers of CU5, being the ones who attend or do not attend GMP, also needed to play a part in determining the quality of GMP activities in their communities. There is evidence suggesting that implementing growth monitoring without linking it to the promotion aspect of GMP is a waste of resources and a loss of opportunities [25]. That being the case, it can also be said that classifying GMP without the inclusion of caregiver barriers and enablers of GMP results in a waste of resources and missed opportunities to improve the quality of GMP activities.

**Figure 2:**
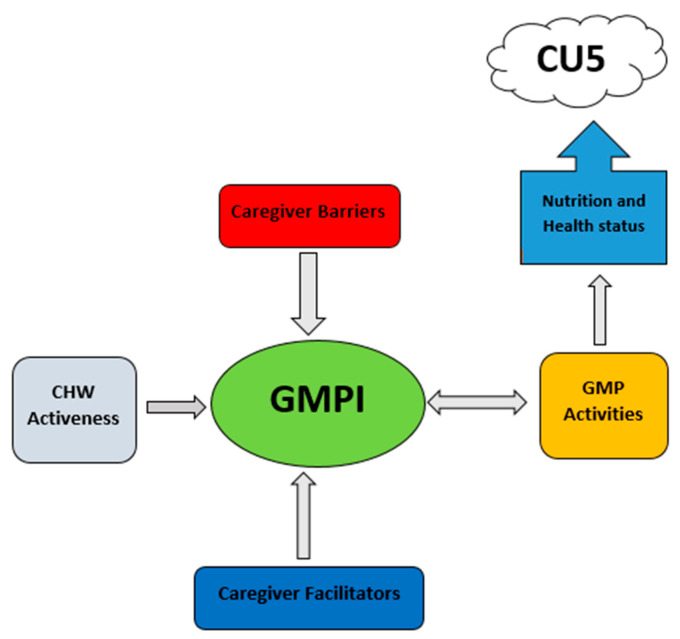
Conceptual framework for improved growth monitoring and promotion.

The construction of the GMPI was guided by the conceptual framework by Ashworth et al. [14] and adapted for this study as shown by Moyo et al. [11] in their protocol paper. It was deemed the best fit to depict the study as a whole. It is from this conceptual framework that the study looked at the different elements that were investigated in phase 2 and now in phase 3, resulting in a new conceptual framework (Figure 2). Based on the results of this study, a new conceptual framework for the GMPI is illustrated below (Figure 2). For the Umguza district, the conceptual framework outlines the actual caregiver barriers that weigh down the GMPI, while the caregiver facilitators push it up. CHW activeness has a direct effect on the GMPI, while GMP activities have an effect and are also affected by the GMPI. All these variables directly impact GMP activities and ultimately affect the child health and nutrition status of CU5. When CHWs are active and caregivers attend GMP activities, this means that the health and nutrition of CU5 are being monitored routinely, and hence both preventive and curative measures can be implemented in a timely manner through improved GMP. This can ultimately result in improved child health and nutrition status of CU5.

This index was developed and validated, as guided by the research protocol [11]. The study identified the different determinants that make up the GMPI. It has been shown that variables Z and Y are based on the magnitude of the barriers and facilitators found/identified, and this has a direct bearing on the GMPI. The more barriers identified (Table 5), the lower the GMPI, while the more facilitators identified (Table 4), the higher the GMPI. The GMPI considers the magnitude of each variable, and therefore it can be applied in any given context. It draws on the characteristics of each variable, no matter the strength or magnitude or lack thereof.

The Delphi Technique outcome used to validate the index did not result in any changes to the new GMPI variables. Contextualisation and validation strengthened the initial acceptance of the GMPI and thus increased its chances of being implemented and resulting in the intended outcomes [26]. The key takeaway is that the GMPI needs to be piloted in other districts; refinements, if any, can be made thereafter.

The researchers, through their scoping literature review [23], showed that not many child health-related indexes exist. Therefore, this new GMPI and its development have added to the body of existing knowledge. Furthermore, the study has important implications for GMP program design as through its use, areas needing allocation of resources (human, financial, time) can be supported appropriately.

### The Implication of the GMPI

The child health indexes that are currently existing are weight-for-height, height-for-age, weight-for-age, and so forth [27]. The variables also follow the names of the indexes as well, i.e., weight, height/length, age. The developed GMPI brings in a new set of variables to assess the quality of GMP activities as conducted by CHWs. The new variables are CHW activeness and barriers and facilitators towards GMP for caregivers. These three variables are very important to the successful implementation of GMP, yet they were never combined into a single index. Thus, the developed GMPI has combined the activeness of CHWs who are the interlink between the communities and the health facilities within the community health system and barriers and facilitators affecting caregivers from attending GMP. The GMPI is at the centre of GMP activities as it can sound alarm bells on the implementation of GMP activities that will be performing poorly. This helps GMP implementers to understand better where the actual problem is when for example the number of children attending GMP is low. The GMPI will also be able to indicate where the challenge is. Is it the CHWs? Is it the caregivers? What is currently existing are indicators that show the number of children whose weight or MUAC has been measured. What these data do not tell us is anything related to whether few children were measured due to the inactivity of CHWs or caregivers not bringing CU5 for GMP. Using the GMPI, it would be clear which GMP aspect is lagging, aiding GMP implementers in developing specific strategies based on the low-performing variable and thus helping to make GMP data more meaningful for all.

To have improved GMP, caregiver barriers need to be reduced, caregiver facilitators need to be increased, and CHWs need to remain active for the GMPI to remain high. Improved GMP will then result in improved nutrition and health status of CU5.

## 5. Recommendations

The study recommendations are outlined as follows for each GMP constituent and are thus for GMP program implementers (MOHCC, NGOs, CBOs, research and funding institutions):

Community Health Workers

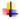
 CHWs are volunteers and are not meant to be working full time, i.e., 40 h a week. Furthermore, this is their understanding when they are enrolled as CHWs. It is thus unclear what the correct position should be as on paper it is said they should work for 2–3 half days a week, while in reality, they are working longer than this. The correct position needs to be established and enforced to address the risk of volunteer burnout and the demise of the active CHW cadre.

Caregivers of children under five years

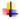
 Strategies need to be put in place to reduce the number of barriers hindering caregivers from attending GMP. The objective of GMP for CU5 is a preventive measure along with providing a curative platform to prevent child morbidity and morbidity. When caregivers do not attend GMP, the children suffer ultimately. It is thus imperative for even policies to be put in place that not only ensure attendance but also address the barriers such as those that were identified by this study.

New GMPI

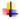
 Based on the outcome of the validation exercise with the technical experts, the GMPI must be piloted in a selected district. That is the only way that GMP implementers can make practical improvements, if any, to improve the index.

### Limitations

The variable of CHW activeness is prone to recall bias as it was based on a recollection of the number of hours per week spent on CHW activities. Only one level of validation could be held for the GMPI with technical experts, and there was nothing for the lower-level end users at the district level due to limitations in funding for the study.

In this study, criterion validity was not considered.

## 6. Conclusions

The development of this GMPI opens doors for further refinement. A new dawn has arrived where public health practitioners are being challenged to continue to identify practical ways in which all the data that are obtained daily from varying health programs become more meaningful to the end users such as the MOHCC. The GMPI will be used to classify the GMP performance of districts through the DHIS2, thus strengthening the quality of GMP activities.

## Figures and Tables

**Table 1:** Technical experts in the Delphi consultations.

Type of Expert	Number
Academia	3
MOHCC	3
Policy development	3
Nutrition program management specialist	3
SBC specialist	3
Total	15

**Table 2:** Summary of merged findings.

Findings from Scoping Literature Review	Findings from CHW Survey	Findings from Barrier Analysis with Caregivers of CU5	Findings from the Merged Analysis
Antecedent factors − Distance and socio-cultural constraints − CHW activeness − Participation of fathers in GMP activities − Poor understanding and interpretation of growth charts − Poor communication between caregivers and CHWs − Full vaccination status and complacency Attributes − Education status of parents, knowledge − Attitude and practices of caregivers Consequences − Timely health interventions, improved child health outcomes − A platform to promote optimal child health practices of GMP activities Characteristics of index development − From the review of the literature, there are no existing indexes that interlink CHW activities and caregiver behaviours towards GMP	Frequency of GMP activities − 68.8% of CHWs conduct GMP activities monthly Background or working environment − 98.4% had a weighing scale − 55.9% had adequate IEC materials such as IYCF counselling cards − Spend an average of 16 h per week on various CHW activities − Support an average of 26 children under five years. Relationship between knowledge of GMP and frequency of activities − There was a significant relationship between the level of knowledge in GMP aspects and the frequency of GMP activities, X^2^ (1, N = 186) = 16.412, *p* = 0.000	Barriers − Distance to health facilities − Time taken during GMP activities − Transport costs − Family support Facilitators − Distance to health facilities	In this study, the GMP index (GMPI) was defined as the quality of GMP activities. The antecedent factor of CHW activeness was explored further through the CHW survey and constitutes variable X of the GMPI. The attribute of attitude and practices of caregivers was explored in further depth through the barrier analysis; 4 barriers and 1 facilitator of caregivers towards GMP attendance were identified and thus constituted variables Y and Z, respectively.

**Table 3:** CHW activeness.

CHW Activeness	Frequency	%	Scores
Least Active (0–8 h per week)	12	6.5	1
Moderately Active (9–16 h per week)	89	47.8	2
Active (17–24 h per week)	66	35.5	3
Very Active (25–32 h per week)	14	7.5	4
Most Active (33–40 h per week)	5	2.7	5

**Table 4:** Facilitators of GMP.

Number of Facilitators	Classification	Score
4 or more	Many	3
2 to 3	Moderate	2
1 or less	Few	1

**Table 5:** Barriers towards GMP.

Number of Barriers	Classification	Score
4 or more	Many	3
2 to 3	Moderate	2
1 or less	Few	1

**Table 6:** GMPI thresholds.

**Threshold**	0–2.3	2.4–4.7	4.8–7
Classification	Low	Medium	High

**Table 7:** Community Health Worker Activeness.

CHW Activeness		Score
Least active	0–8 h	1
Moderately active	9–16 h	2
Active	17–24 h	3
Very active	25–32 h	4
Most active	33–40 h	5

**Table 8:** Caregiver facilitators of GMP.

Number of Facilitators	Classification	Score
4 or more	Many	3
2 to 3	Moderate	2
1 or less	Few	1

**Table 9:** Caregiver barriers to GMP.

Number of Barriers	Classification	Score
4 or more	Many	3
2 to 3	Moderate	2
1 or less	Few	1

**Table 10:** GMPI classification.

**Threshold**	0–2.3	2.4–4.7	4.8–7
Classification	Low	Medium	High

## Data Availability

Data will be made available upon reasonable request.
